# Comprehensive analysis of cuproptosis in immune response and prognosis of osteosarcoma

**DOI:** 10.3389/fphar.2022.992431

**Published:** 2022-10-03

**Authors:** Mingzhe Li, Qiang Song, Yunfeng Bai, Feng Hua, Tao Wu, Jun Liu

**Affiliations:** ^1^ Department of Orthopedics, The Second Affiliated Hospital of Nanjing Medical University, Nanjing, China; ^2^ Department of Urology, The First Affiliated Hospital of Nanjing Medical University, Nanjing, China

**Keywords:** copper-induced cell death, cuproptosis, osteosarcoma, immunotherapy, tumor microenvironment

## Abstract

Copper-induced cell death, a form of apoptosis, has been extensively investigated in human diseases. Recent studies on the mechanisms underlying copper-induced cell death have provided innovative insights into copper-related toxicity in cells, and this form of programmed cell death was termed cuproptosis. Herein, we conducted a comprehensive analysis to determine the specific role of cuproptosis in osteosarcoma. Using consensus clustering analysis, patients with osteosarcoma from the TARGET database were classified into subgroups with distinct cuproptosis-based molecular patterns. Accordingly, these patients displayed diverse clinicopathological features, survival outcomes, tumor microenvironment (TME) characteristics, immune-related scores, and therapeutic responses. Furthermore, we constructed a cuproptosis-based risk signature and nomogram, as well as developed a cuproptosis score for improved patient characterization. The prognostic model and cuproptosis score were well validated and confirmed to efficiently distinguish high- and low-risk patients, thereby affording great predictive value. Finally, we verified the abnormal expression of prognostic CUG in OS patients by immunohistochemistry. In conclusion, we suggest that cuproptosis may play an important role in regulating the tumor microenvironment features, tumor progression and the long-term prognosis of osteosarcoma.

## Introduction

Osteosarcoma (OS) is the most common primary bone tumor in children and adolescents (Longhi et al., 2006; Kumar et al., 2018). OS is characterized by high malignancy and aggressiveness, poor prognosis and high disability rate (Ritter and Bielack, 2010). OS metastases, especially lung metastases, are the most common cause of cancer-related death (Huang et al., 2019). Despite advances in combination therapy (surgery, chemotherapy, neoadjuvant chemotherapy, radiotherapy, and immunotherapy), the 5-years survival rate of patients with OS, especially those with metastatic and recurrent disease, is still far from satisfactory (Dyson et al., 2019; Gill and Gorlick, 2021). Although many genes have been identified as potential biomarkers for the prediction and treatment of OS (Brown et al., 2017; Lyskjær et al., 2022). But the complexity and instability of the OS genome have hindered the progress of treatment. Therefore, a better understanding of the mechanisms underlying OS tumorigenesis and progression is urgently needed to identify more effective and specific biomarkers for early prediction, survival assessment, and treatment.

Several scheduled and precisely controlled programmed cell death events, including apoptosis, necroptosis, pyroptosis, and ferroptosis, are well-known to occur during the development of multicellular organisms (Wang et al., 2022). Recently, Tsvetkov et al. have reported a new strategy to regulate cell death, which differs from known cell death mechanisms. Copper ions can induce cell death by blocking a known cell death mechanism. This copper-induced cell death mechanism involves protein lipoylation and is termed “cuproptosis” (Tsvetkov et al., 2022). Further, this study identified seven genes (FDX1, LIAS, LIPT1, DLD, DLAT, PDHA1, PDHB) that promote cuproptosis, as well as three genes (MTF1, GLS, CDKN2A) that inhibit this mechanism. Cuproptosis is a new research direction. So far, many studies have shown that cuproptosis plays a role in tumorigenesis and progression of various cancers, including melanoma, hepatocellular carcinoma, clear cell renal cell carcinoma, glioma, and lung adenocarcinoma (Zhang et al., 2022a; Zhang et al., 2022b; Lv et al., 2022; Yun et al., 2022). Moreover, cuproptosis related genes or pathways have been shown to be potential targets for cancer treatment (Mo et al., 2022). However, reports on the underlying mechanisms of cuproptosis in OS are currently unavailable. Considering the susceptibility of various cancers to cuproptosis, we propose that cupropsis may also play an irreplaceable role in OS.

In the present study, we conducted a comprehensive online analysis of the association between cuproptosis-related genes (CUGs) and OS. We further investigated the significance of cuproptosis in OS using genotyping, survival analysis, and immune-related assessments. Moreover, a cuproptosis score was established to assess long-term prognosis and treatment response in patients with OS. Finally, we verified the abnormal expression of CDKN2A in OS tissues by immunohistochemistry. Herein, we, for the first time, propose utilizing the cuproptosis score to quantify the cuproptosis pattern of each patient with OS based on the expression profile of CUGs. This scoring mechanism can help oncologists develop more effective and personalized therapeutic strategies.

## Materials and methods

### Data sourcing and preprocessing

Normalized data of gene expression (in the format of FPKM) and corresponding clinicopathological information of patients with OS (*n* = 88) were collected from TARGET datasets in the UCSC Xena website (https://xena.ucsc.edu/). Among these, patients with missing critical clinical or survival parameters were excluded and finally 86 samples were included for further analysis. The FPKM values were normalized to transcripts per kilobase million (TPM) for further analysis. In addition, OS patients from the GSE21257 dataset (*n* = 54) of the GEO database (https://www.ncbi.nlm.nih.gov/geo/) were used as a validation group. These 10 CUGs were collected from the latest report, as mentioned above. The raw reads of the above data were processed and normalized in R software.

### Consensus clustering analysis

Based on the expression profiles of 10 CUGs from the TARGET dataset, the R package “ConsensusClusterPlus” was applied for consensus unsupervised clustering analysis, and patients with OS from TARGET database were classified into distinct molecular subgroups. The *k*-means algorithm was employed to determine the optimal grouping number. T-SNE was used to verify clusters based on the expression profiles of these genes.

### Association between cuproptosis patterns with the clinical characteristics and prognosis of OS

To assess the clinical significance of distinct cuproptosis patterns, we compared the associations among cuproptosis patterns, clinicopathological features, and survival outcomes in OS. The Kaplan-Meier method was used to assess overall survival between different groups. Gene set variation analysis (GSVA) using the “GSVA” R package was performed to identify differences in biological functions among distinct cuproptosis patterns.

### Identification of differentially expressed genes (DEGs)

The “limma” package in R was used to identify DEGs between distinct molecular subgroups from CUG clustering. Genes with an adjusted *p*-value < 0.05 and |log2(FC)| > 1.0 were considered differentially expressed. Finally, 1708 genes were characterized; among them, 276 DEGs were predicted to be prognostic genes and subjected to further analysis.

### Construction of the risk signature and nomogram

The weighted coefficients were calculated using the least absolute shrinkage and selection operator (LASSO) Cox regression analysis, and the “glmnet” package in R was used to construct a risk model. Patients were divided into high- and low-risk groups according to the optimal cutoff of the risk score, and the survival rate of patients was analyzed using the Kaplan-Meier method. The receiver operating characteristic (ROC) curves were depicted using the “survival-ROC” package in R, and the area under the curve (AUC) was calculated to determine the sensitivity of the survival analysis. Finally, the risk core and clinical factors were integrated into the nomogram using a multivariate Cox regression analysis.

### Construction of the cuproptosis score

Cuproptosis scores were calculated to quantify the cuproptosis patterns for each patient with OS. The Boruta algorithm was used to reduce the size of cuprotosis gene signatures A and B, and principal component 1 was extracted using principal component analysis (PCA) as the signature score. The score for each patient was calculated as follows: cuproptosis score = ∑PC1A-∑PC1B, where PC1A represents the first component of feature A and PC1B represents the first component of feature B. The Kruskal-Wallis test was used to assess the cuproptosis scores of molecular patterns or gene clusters.

### Clinical significance and drug sensitivity of the prognostic cuproptosis score

The chi-square test was used to investigate the relationship between the cuproptosis score and clinical characteristics (age, sex, metastasis, primary tumor site, and state of survival). In addition, we stratified the predictive ability of the cuproptosis score according to distinct clinical characteristics. The ESTIMATE method was used to generate stromal and immune scores to evaluate the tumor purity and distribution of cell types in the tumor microenvironment (TME). The Genomics of Drug Sensitivity in Cancer (GDSC; https://www.cancerrxgene.org/) database was used to predict patient responses to several drugs. The “pRRophetic” package in R was used to quantify IC50 values. Drugs interacting with prognostic CUGs were obtained from the Drug Gene Interaction Database (DGIdb; https://dgidb.genome.) and their 3D structures were visualized using the PubChem website (https://pubchem.ncbi.nlm.nih.gov/).

### Clinical specimens

Tumour tissues and their adjacent normal tissue were obtained from patients who were diagnosed with OS and had undergone surgery in The Second Affiliated Hospital of Nanjing Medical University between 2019 and 2022. The follow-up deadline was June 2022. All patients signed informed consent before using clinical materials. The use of tissues for this study was approved by the ethics committee of the Second Affiliated Hospital of Nanjing Medical University.

### Hematoxylin–eosin (HE) staining and immunohistochemistry (IHC)

Hematoxylin-eosin staining and immunohistochemistry were performed after tissue fixation and paraffin embedding. Slides were cut to 5 μm wide, dewaxed, and rehydrated. Endogenous peroxidase was inactivated with 3% H2O2 for 10 min and then incubated with 5% bovine serum albumin (BSA) blocking solution for 1 h at room temperature. Sections were incubated with the corresponding protein antibodies overnight at 4°C. The antibodies used are as follows: the Ki67 (Cat. No. GB111499, 1:500), PCNA (Cat. No. GB11010, 1:500), and CDKN2A (Cat. No. GB111143, 1:1,000) antibodies were purchased from Servicebio (Wuhan, China); The sections were then incubated with biotinylated goat anti-rabbit secondary antibody for 30 min at 37°C. The newly prepared 3,3 ′-diaminobenzidine (DAB) reagent (Servicebio, Wuhan, China) was used for color development.

### Statistical analysis

All statistical analyses were performed using R 4.1.0. The experimental results were expressed as mean ± SD (standard deviation), and one-way ANOVA was used to determine statistical significance. Statistical significance was set at a two-sided *p*-value < 0.05. All experiments were repeated more than three times.

## Results

### Survival analysis of OS patients based on CUGs

**FIGURE 1:**
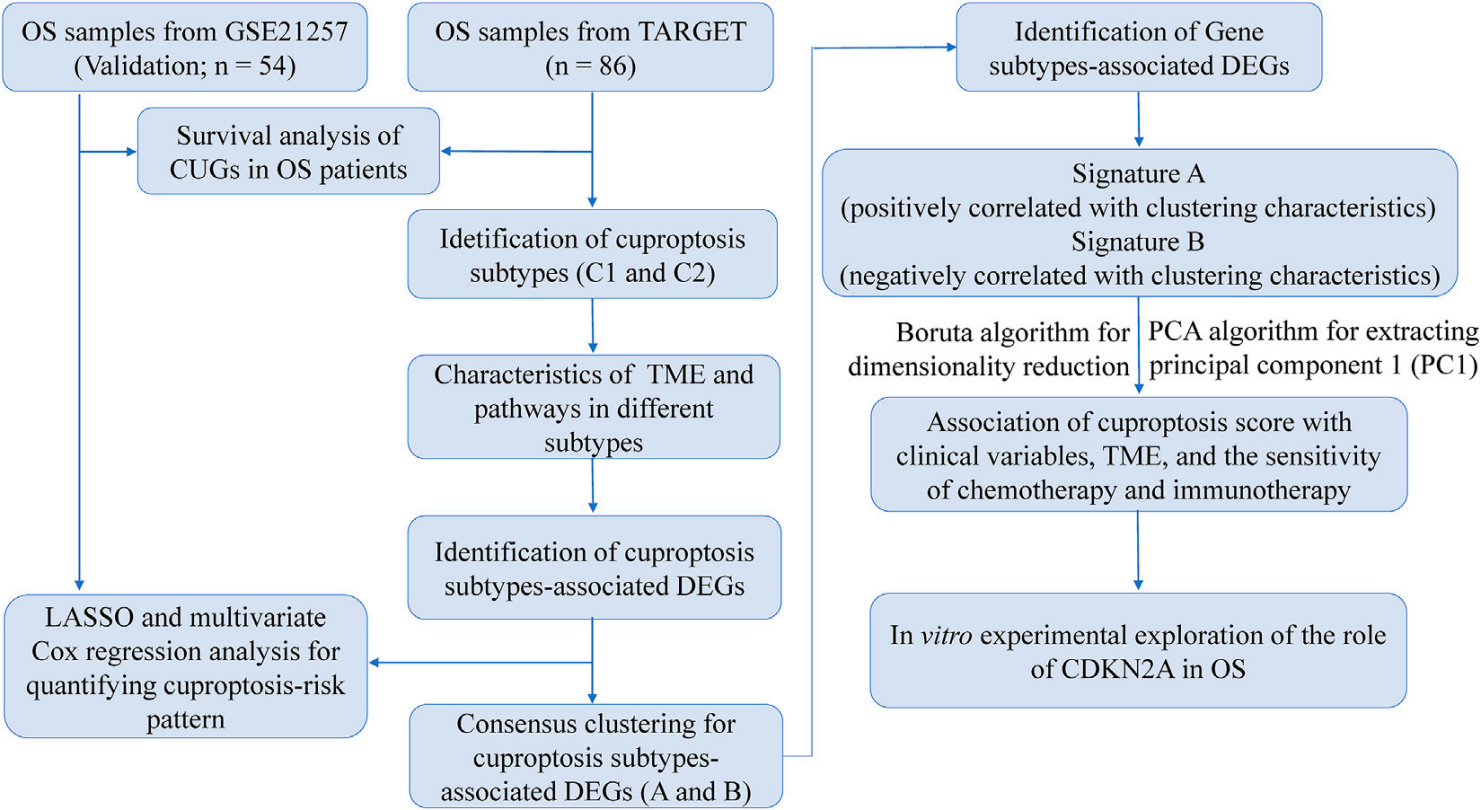
A flow chart showing the details of the analysis.

**FIGURE 2:**
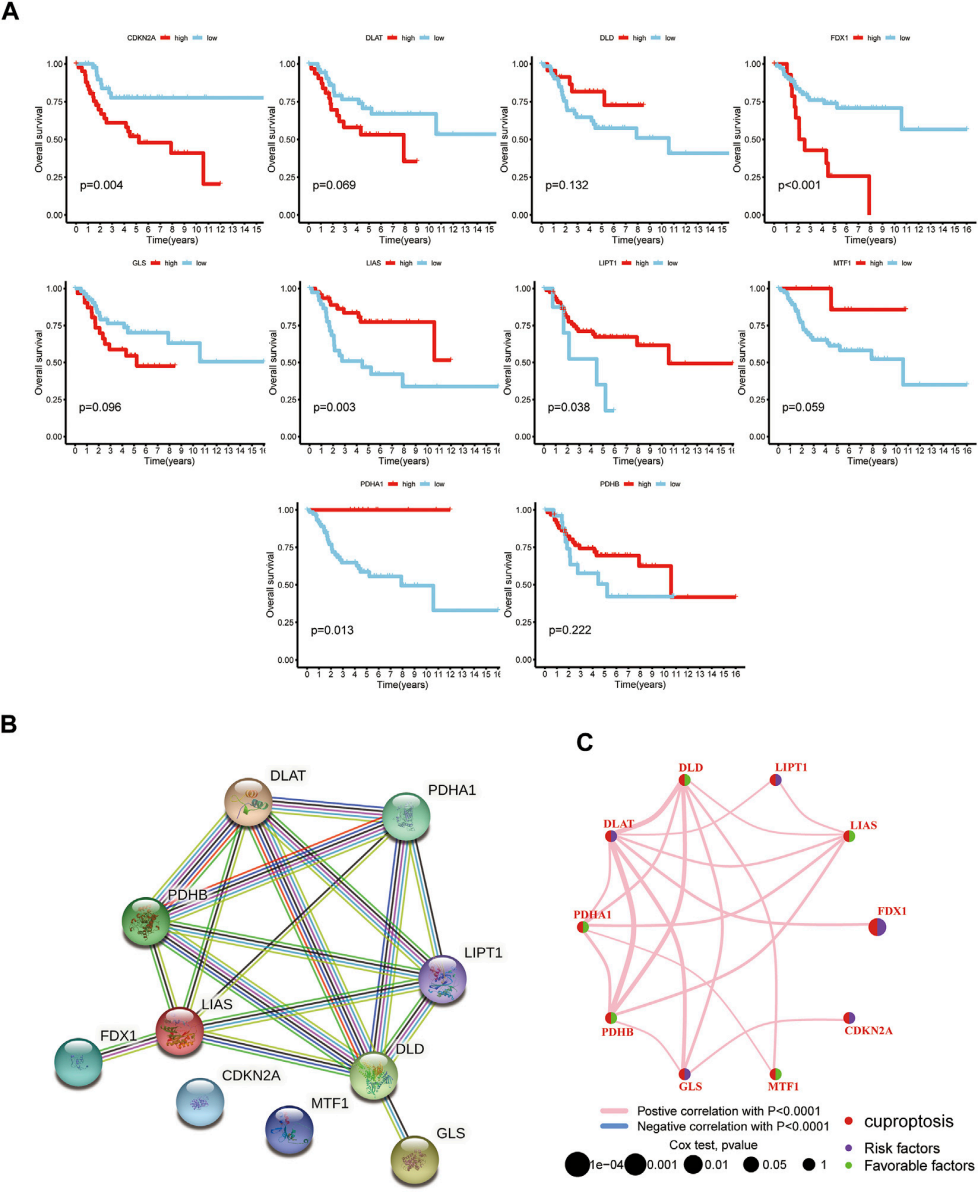
The prognostic value of 10 CUGs in patients with OS. **(A)** Kaplan-Meier curves for the 10 CUGs in OS patients from TARGET database. **(B)** The PPI network acquired from the STRING database among the CUGs. **(C)** A network of correlations including CUGs in the TARGET cohort. (*p* < 0.05 *; *p* < 0.01 **; *p* < 0.001 ***).

To explore the association of CUGs with OS prognosis, we performed a survival analysis for each CUG for patients with OS from the TARGET database. It is well-established that high levels of CDKN2A and FDX1 are significantly associated with worse overall survival in patients with OS, whereas LIAS, LIPT1, MTF1, and PDHA1 displayed the opposite trend (*p* < 0.05) (Figures 1A–2A). Subsequently, the survival impact of CUGs was verified by GSE21257 dataset (Supplementary Figure S1). Furthermore, Figure 2B illustrates the significance of these regulators in the OS cohort and their interactions based on the protein-protein interaction analysis of the STRING website. Figure 2C presents the network, regulatory relationships, and survival significance of CUG interactions in patients with OS.

### Construction of cuproptosis subtypes in OS patients

**FIGURE 3:**
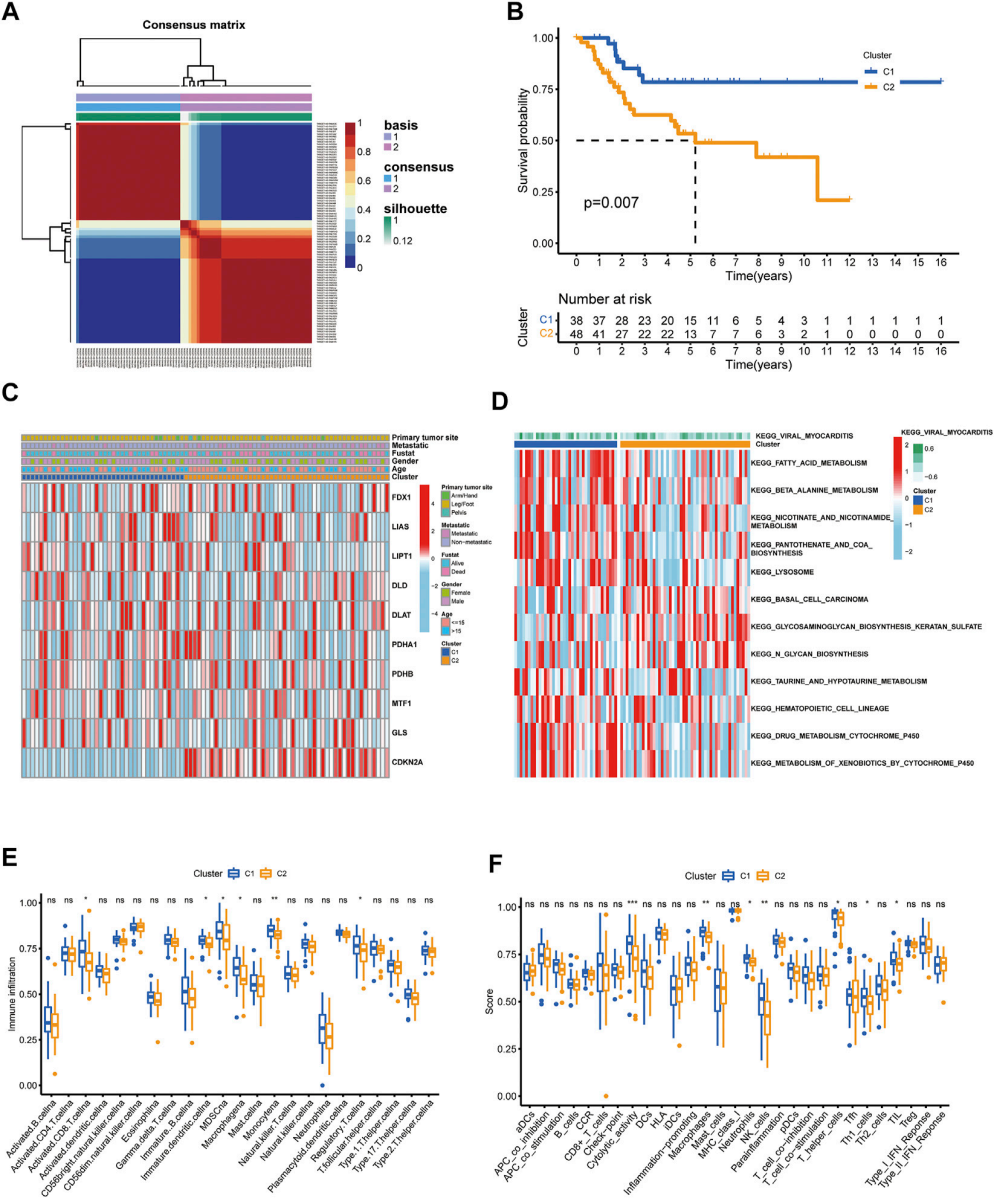
Identification of cuproptosis subtypes in OS. **(A)** t-SNE of the mRNA expression profiles of CUGs from the OS samples in the TARGET dataset confirmed the two clusters: C1 and C2. **(B)** Kaplan-Meier curves for the two molecular patterns of OS patients. **(C)** Heatmap depicted the correlation between the subtypes and different clinicopathological characteristics. **(D)** GSVA enrichment analysis of biological pathways between the two distinct subtypes. Boxplots showed abundance of 23 infiltrating immune cell types **(E)** and differences in immune scores **(F)** in the two cuproptosis subtypes. (*p* < 0.05 *; *p* < 0.01 **; *p* < 0.001 ***).

Unsupervised nonnegative matrix factorization clustering was performed on OS samples from TARGET database based on the mRNA expression levels of 10 CUGs. Molecular clustering effects and cophenetic correlation analyses were used to determine the optimal k. We examined k from 2 to 5, and among all k values, k = 2 was most suitable for clustering, overall survival analysis, and contours (Figures 3A,B; Supplementary Figures S2A,B). The two subgroups were named C1 and C2 (*p* < 0.05 was considered statistically significant). As shown in Figure 3C, comparing gene expression and clinical variables between the two clusters revealed significant differences in CUG expression and clinical characteristics. Especially in C2, a subgroup with poor prognosis, CDKN2A showed significantly higher expression, which was consistent with the results of previous survival analysis. In addition, the C2 group mainly included patients younger than 15 years of age. We propose two guesses. One is that OS patients younger than 15 years of age have a worse prognosis, which is consistent with previous reports (Janssen et al., 2021). However, more and more studies have shown that the age of OS patients has no significant correlation with the prognosis (Eleutério et al., 2015). The second is that patients younger than 15 years of age with high CDKN2A expression have a worse prognosis. However, there is no conclusive study to confirm this conjecture. GSVA analysis showed that Cluster two was significantly enriched in tumor-related pathways, while Cluster one was mainly enriched in metabolism-related pathways (Figure 3D). In addition, we examined the effect of cuproptosis on the TME. The results revealed that the C1 group exhibited increased lymphocyte infiltration and a high immune score (Figures 3E,F). (*p* < 0.05 was considered statistically significant).

### Construction of a DEG-based prognostic model from cuproptosis subtypes

**FIGURE 4:**
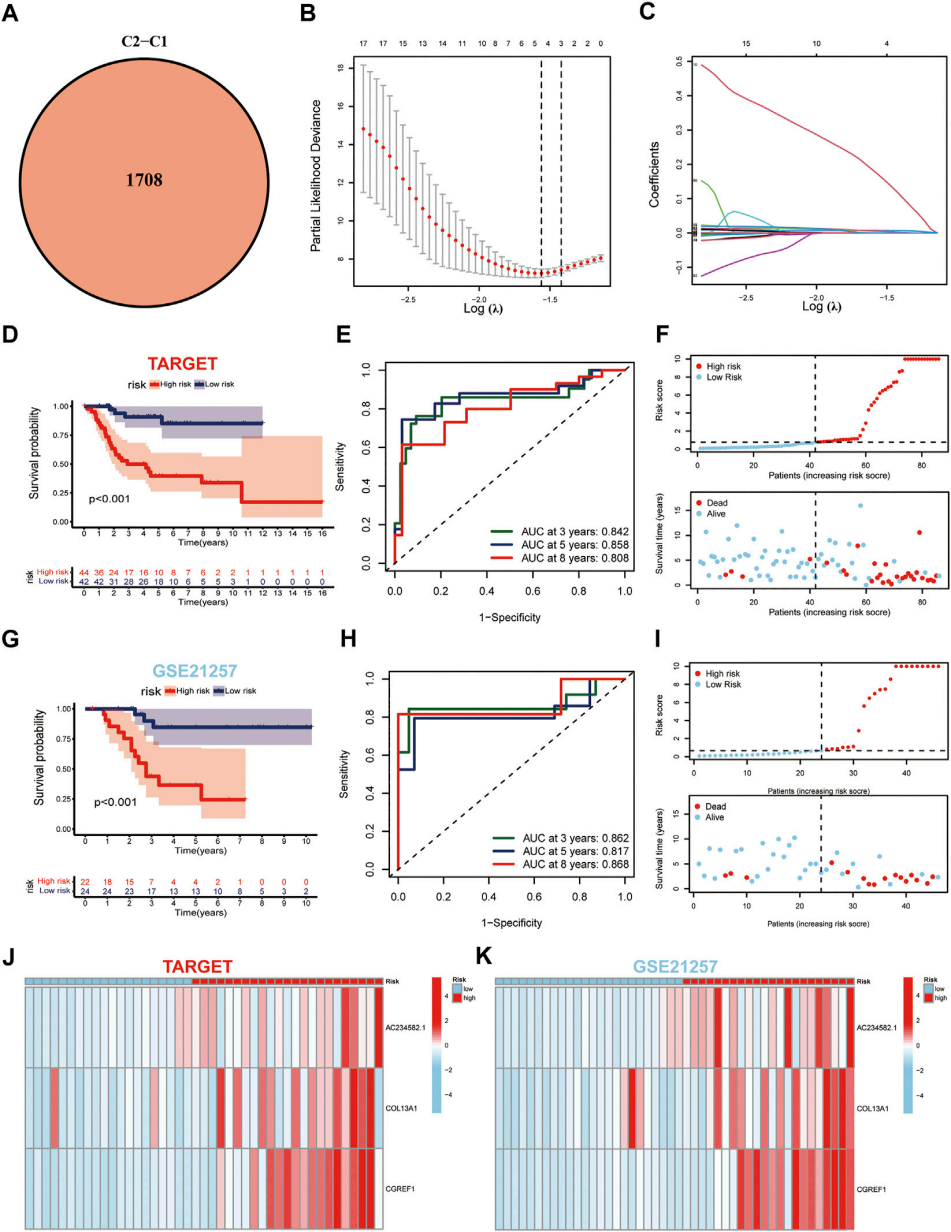
Generation and validation of the prognostic model. **(A)** Venn diagram of the DEGs *via* pairwise comparison among two subgroups. **(B,C)** LASSO regression analysis used to construct the prognostic model. **(D)** Survival analysis in the TARGET-OS cohort. **(E)** ROC curves for the predictive survival in TARGET-OS cohort. **(F)** Ranked dot and scatter plots showing the risk score distribution and patient survival status in TARGET-OS cohort. **(G–I)** Validation of prediction results from GSE21257 dataset against TARGET database. **(J,K)** Expression patterns of three selected prognostic genes in high-and low-risk groups.

Considering the non-negligible biological phenotypic differences between the two cuproptosis subgroups, we used intergroup differential genes to determine the prognostic benefit for patients with OS. First, as described above, 1708 DEGs were generated between the two subgroups (Figure 4A). Among these, 276 DEGs were predicted to be prognostic genes and were subjected to further analysis (Supplementary Table S1). In addition, we plotted the enrichment analysis results of 276 DEGs, including Gene Ontology and Kyoto Encyclopedia of Genes and Genomes (Supplementary Figures S3A,B). Subsequently, these prognostic genes were subjected to LASSO regression analysis to construct the risk signature, and the risk score for each patient with OS was calculated using the following formula: Risk Score = 0.5797*AC234582.1+ 0.0145*COL13A1+ 0.0298*CGREF1 (Figures 4B,C). Furthermore, Kaplan-Meier analysis in the TARGET cohort indicated that high-risk patients had apparently better OS compared to the low-risk groups (*p* ≤ 0.01) (Figure 4D). Besides, the model was well validated as the ROC curves showed, among which the TARGET cohorts obtained AUC of 0.842, 0.858, and 0.808 at 3-years, 5-years, and 8-years, respectively (Figure 4E). The risk plots of risk score showed that with the increase of risk score, OS time decreased, while mortality increased, and the most significantly prognostic genes were shown in the heatmap (Figures 4F,J). The GSE21257 dataset again proved the accuracy of the prediction results with the TARGET database (Figures 4G–I,K).

### Construction of a nomogram to predict patient prognosis

**FIGURE 5:**
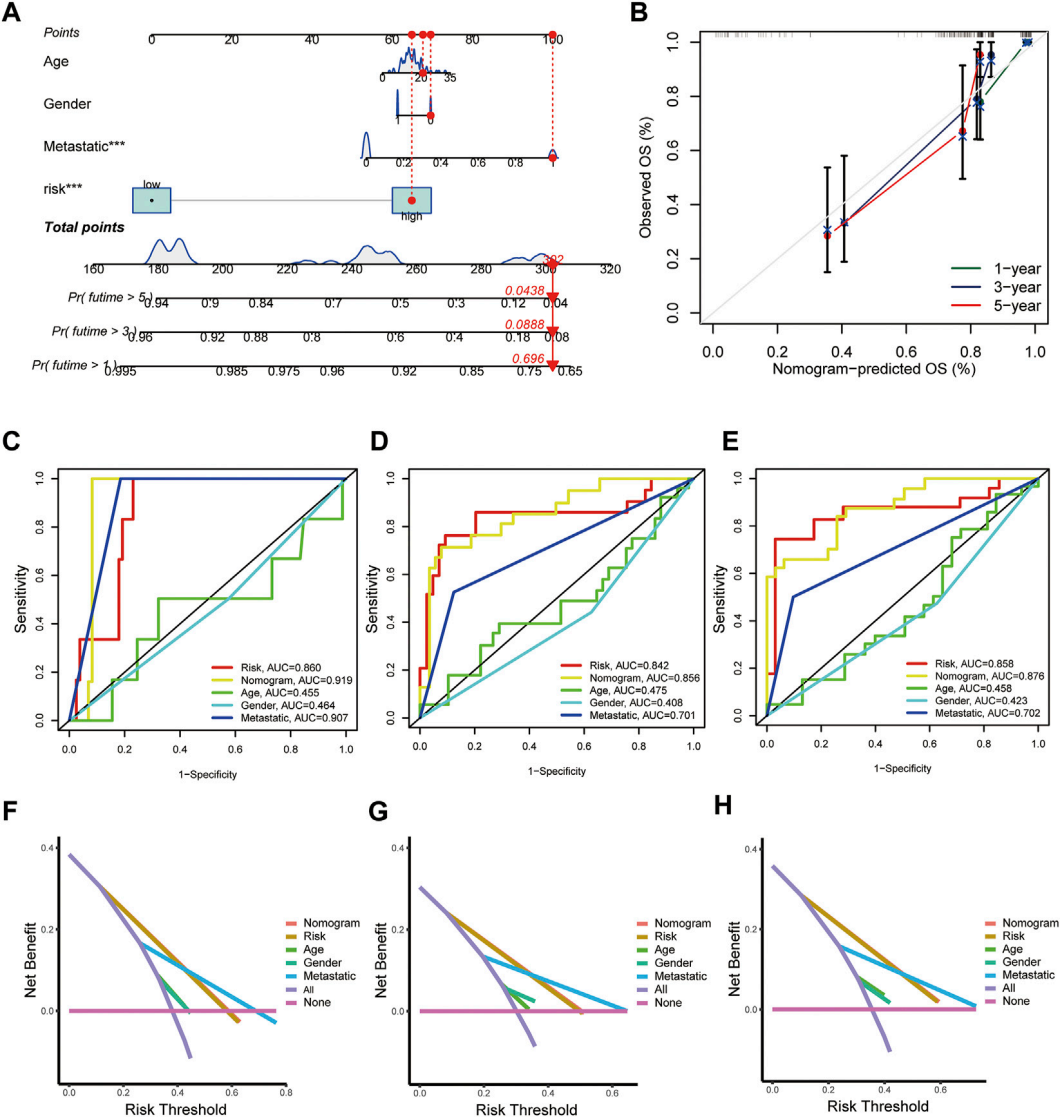
Construction and validation of a nomogram for predicting the prognosis of OS patients. **(A)** Nomogram for predicting the 1-, 3-, and 5-years OS of osteosarcoma patients in the TARGET-OS cohort. **(B)** Calibration curves for validating the established nomogram. **(C–E)** The ROC curves of the nomograms compared for 1-, 3-, and 5-years OS in osteosarcoma patients, respectively. **(F–H)** The DCA curves of the nomograms compared for 1-, 3-, and 5-years OS in osteosarcoma patients, respectively. (*p* < 0.05 *; *p* < 0.01 **; *p* < 0.001 ***).

Next, the nicely established prognostic model was depicted in a nomogram (Figure 5A), and the calibration curve displayed a certain concordance between the estimated survival probability and observed results for 3-, 5-, and 8-years overall survival (Figure 5B). Moreover, the comparative ROC curves revealed that the nomogram was superior to any clinical factor alone in predicting the long-term survival of patients (Figures 5C–E). In addition, the DCA curves of the nomograms could evaluate the accuracy of the model. According to Figures 5F–H, the score curves of the model and risk score were the highest, indicating that the model and risk scores had better predictive ability than other clinical factors.

### TME characteristics of risk subtypes

**FIGURE 6:**
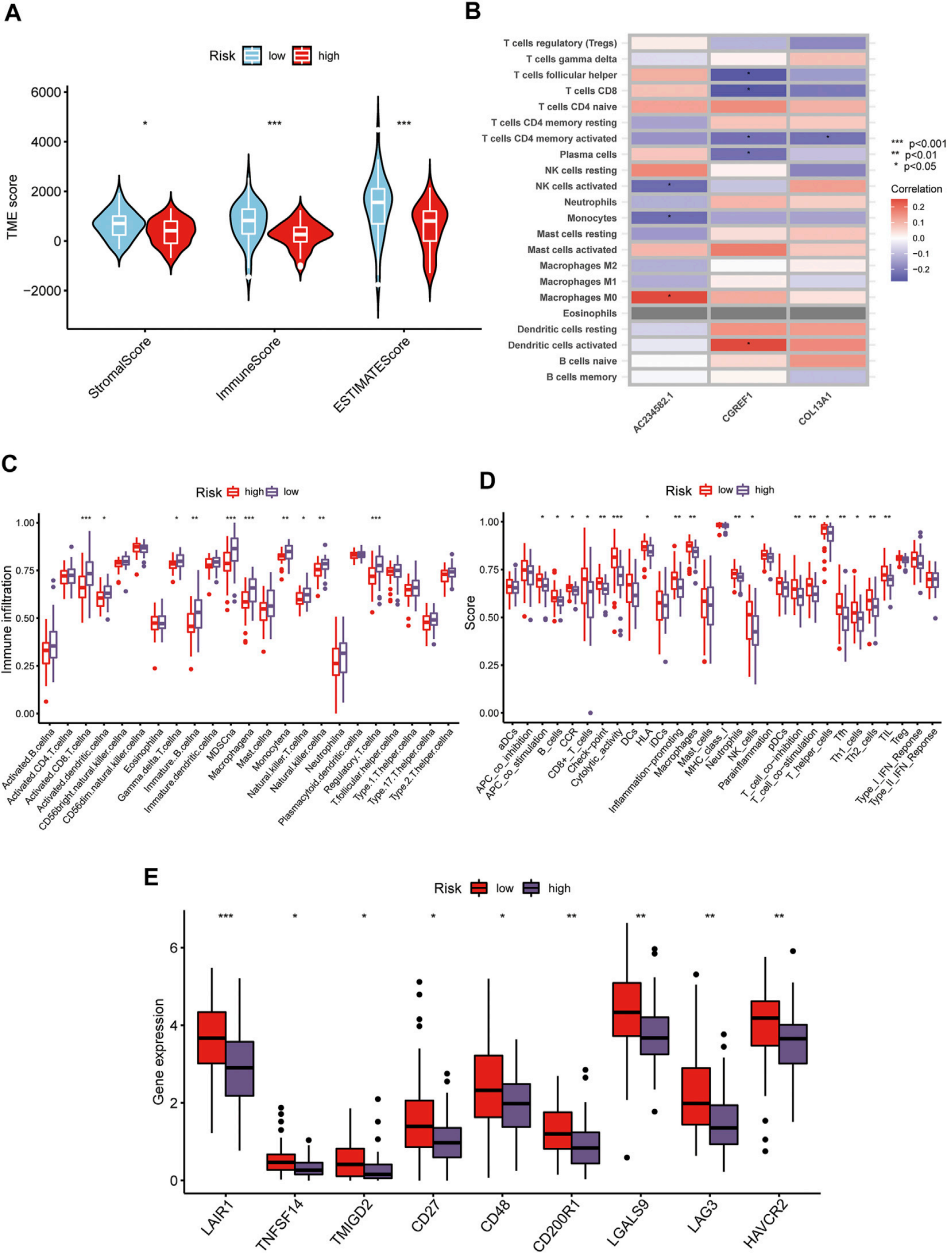
Distinct TME characteristics and mutation of OS patients according to the risk score. **(A)** Correlations between risk score and both immune and stromal scores. **(B)** Correlations between risk score and immune cells. Boxplots showed **(C)** abundance of 23 infiltrating immune cell types and **(D)** differences in immune scores in the two risk score groups. **(E)** The boxplot shows variations in the expression of CUGs between the two risk score groups. (*p* < 0.05 *; *p* < 0.01 **; *p* < 0.001 ***).

The CIBERSORT algorithm was used to evaluate the correlation between risk score and immune cell abundance. As shown in Figure 6A, the risk score was positively correlated with the matrix and immune scores. Subsequently, we explored the correlation between the selected prognostic marker genes and immune cell enrichment. We concluded that several immune cells are closely related to the selected genes, such as macrophages M0 and activated dentritic cells (Figure 6B). Additionally, the low-risk group had higher immune cell infiltration and immune function scores than the high-risk group (Figures 6C,D). Finally, we evaluated the relationship between immune checkpoints (ICPs) and risk scores. As shown in Figure 6E, the expression of the nine ICPs differed between the two risk subgroups.

### Generation and TME characteristics of patient subgroups based on DEGs clustering

**FIGURE 7:**
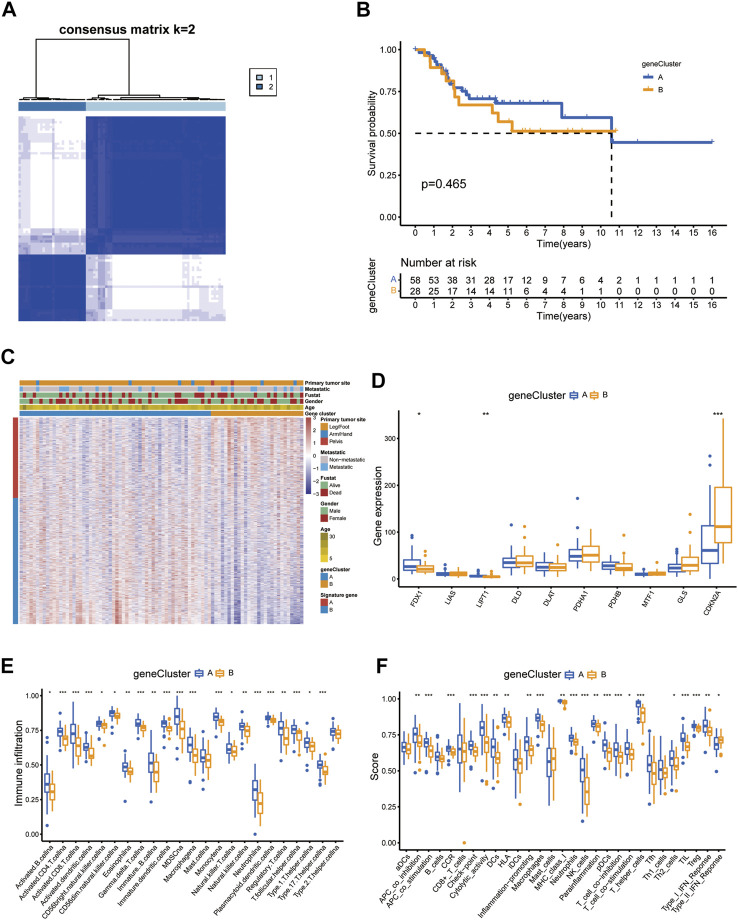
Prognosis and TME characteristics in two cuproptosis gene clusters for OS patients. **(A)** Consensus matrix heatmap defining two gene clusters according to the prognostic DEGs. **(B)** Kaplan-Meier survival analysis for patients in the two gene clusters. **(C)** Clinical features of the two gene clusters. **(D)** The boxplot shows variations in the expression of CUGs between the two gene clusters. Boxplots showed **(E)** abundance of 23 infiltrating immune cell types and **(F)** differences in immune scores in the two gene clusters. (*p* < 0.05 *; *p* < 0.01 **; *p* < 0.001 ***).

Similar to the CUG-based grouping of patients with OS, we subdivided the patients into two groups (designated as A and B; NA = 58, NB = 28) using consensus clustering analysis based on the gene clustering of prognostic DEGs in Supplementary Table S1 (Figure 7A; Supplementary Figures S4A,B). Dimensionality reduction was proposed by the Boruta algorithm based on gene signatures A and B. The heatmap depicted the 1708 most abundant DEGs identified in the geneClusters. In addition, we could also conclude from the heatmap that OS patients younger than 15 years old were concentrated in geneCluster B. The following survival analysis showed that patients in gene-cluster B had a worse prognosis than those in gene-cluster A, which was consistent with the previous analysis. But its significance *p* value was above 0.05 (Figures 7B,C). Besides, the differential expression of FDX1, LIPT1, and CDKN2A between the two gene-clusters was statistically significant, indicating that these genes were closely related to the tumorigenesis and progression of osteosarcoma, which was consistent with the expected results of the cuproptosis patterns (Figure 7D). Similarly, the TME characteristics of each gene cluster were displayed as immune cell infiltration (Figure 7E) and immune-related function analyses (Figure 7F). As the results indicated, nearly all types of immune-cells and all processes of immune-related function turned out to be significantly different between the two gene-clusters.

### Establishment and validation of the cuproptosis score in OS patients

**FIGURE 8:**
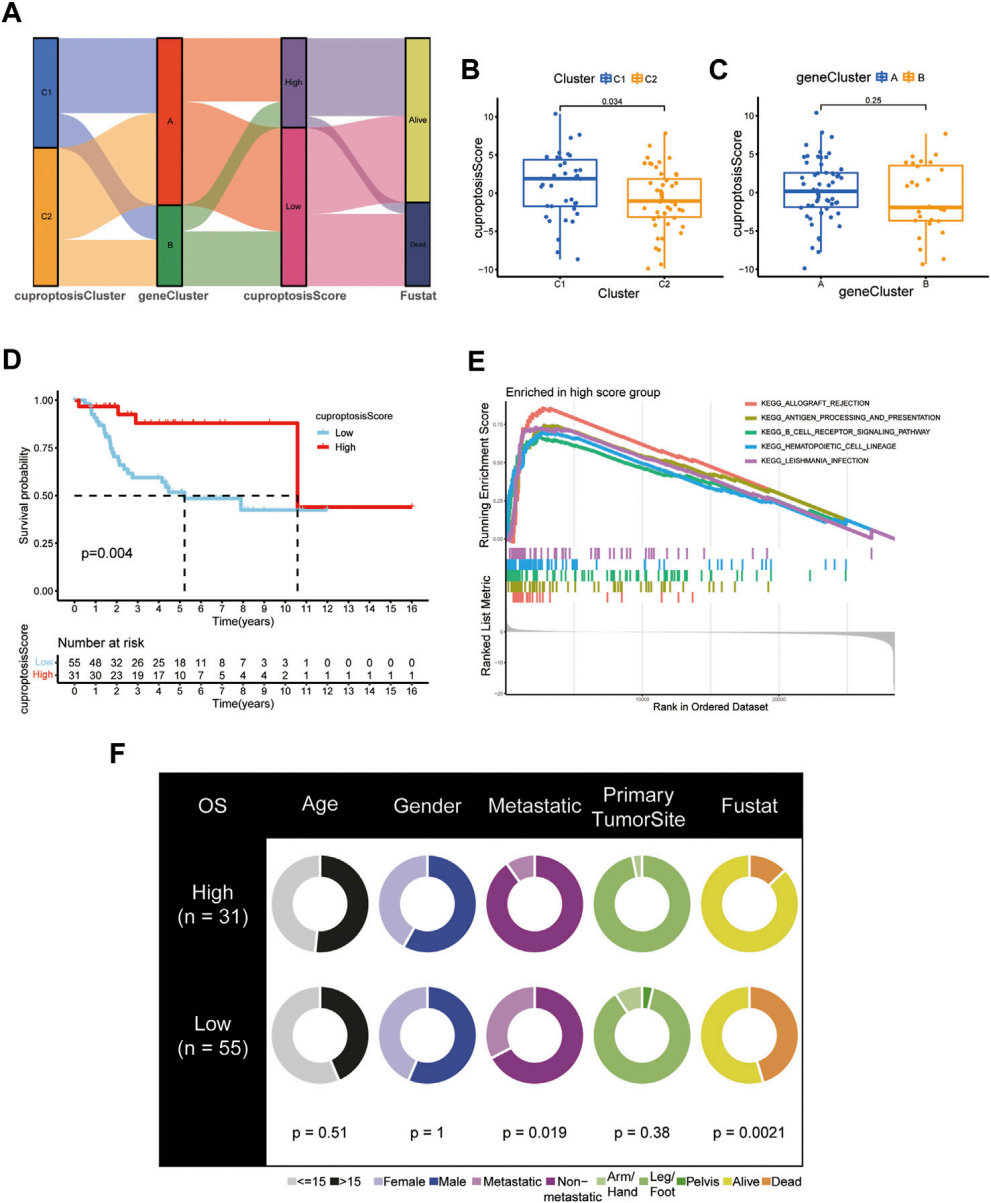
Development and validation of the cuproptosis scoring system for OS. **(A)** Sankey Diagram of cuproptosis clusters, gene clusters, cuproptosis score, and clinical outcomes. Differences in cuproptosis score between **(B)** the two cuproptosis subtypes and **(C)** the two gene clusters. **(D)** Kaplan-Meier analysis of the OS between the two cuproptosis score groups. **(E)** GSEA showed the different pathways significantly enriched in the high score group. **(F)** Clinical characteristics for the high and low cuproptosis score groups. (*p* < 0.05 *; *p* < 0.01 **; *p* < 0.001 ***).

Based on 276 prognostic DEGs, we used PCA to calculate the cuproptosis score for each patient with OS. Figure 8A presents the distribution of patients with OS in the cuproptosis clusters, gene clusters, and cuproptosis scoring system, as well as the survival status. Additionally, reverse validation was performed, and the plots revealed that cuproptosis could discriminate between cuproptosis and gene clusters (*p* < 0.05) (Figures 8B,C). Subsequently, based on the calculated cuproptosis score, each patient in the validation cohort was analyzed for survival probability, and the results revealed that a higher cuproptosis score was associated with a significantly improved probability of patient survival (Figure 8D). Moreover, we sought to reveal the potential vital biological processes associated with cuproptosis scoring system. The results of GSEA analysis showed that in high-score group, immune-related pathways were enriched (Figure 8E). These results indicated that patients in the high-score group may have a more efficient immune response and a better immunotherapy response. Moreover, our results revealed that the cuproptosis score was significantly associated with survival status and metastasis in patients with OS; however, this association was not observed with other clinical factors such as age, sex, and primary tumor site (Figure 8F; Table 1). (*p* < 0.05 was considered statistically significant).

**TABLE 1:** Relationship between cuproptosis score and clinicopathological features of OS in TARGET.

Covariates	Total	High	Low	*p* Value
		No. (%)	No. (%)	
Age (years)				0.51
≤15	46 (53.49%)	15 (48.39%)	31 (57.41%)	
>15	39 (46.51%)	16 (51.61%)	24 (43.64%)	
Gender				1
Female	37 (43.02%)	13 (41.94%)	24 (43.64%)	
Male	48 (56.98%)	18 (58.06%)	31 (56.36%)	
Status				0.0021
Alive	56 (66.28%)	27 (87.1%)	30 (54.55%)	
Dead	29 (33.72%)	4 (12.9%)	25 (45.45%)	
Metastatic				0.019
Metastatic	21 (24.42%)	3 (9.68%)	18 (32.73%)	
Non-metastatic	65 (75.58%)	28 (90.32%)	37 (67.27%)	
Primary tumor -site				0.38
Arm/Hand	6 (6.98%)	1 (3.23%)	5 (9.09%)	
Leg/Foot	78 (90.7%)	30 (96.77%)	48 (87.27%)	
Pelvis	2 (2.33%)	0 (0%)	2 (3.64%)	

### Indicative role of cuproptosis score in TME, immunotherapy, and chemotherapy of OS

**FIGURE 9:**
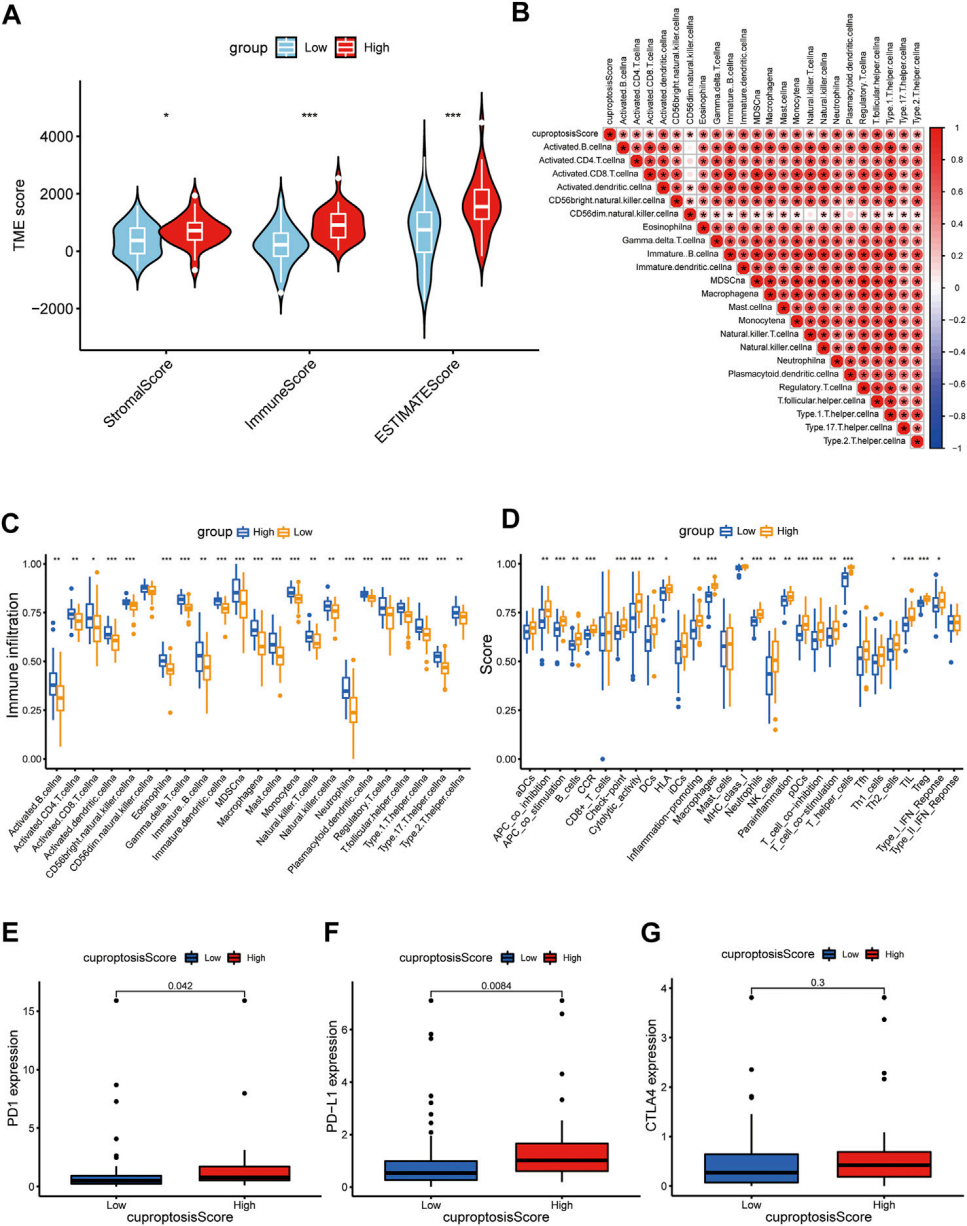
Distinct TME characteristics and mutation of OS patients according to the cuproptosis score. **(A)** Correlations between cuproptosis score and both immune and stromal scores. **(B)** Correlations between cuproptosis score and immune cells. Boxplots showed **(C)** abundance of 23 infiltrating immune cell types and **(D)** differences in immune scores in the two cuproptosis score groups. **(E–G)** The boxplots showed variations in the expression levels of PD1, PD-L1, and CTLA4 between the two cuproptosis score groups. (*p* < 0.05 *; *p* < 0.01 **; *p* < 0.001 ***).

The TME status is another critical factor that impacts the level of immune cell infiltration and immunotherapy treatment response in cancer. Therefore, we applied the ESTIMATE algorithm to evaluate the association of the cuproptosis score with the TME from three perspectives: stromal score, immune score, and ESTIMATE score. The cuproptosis score was superior in all three immune groups (*p* < 0.05) (Figure 9A). Correlation analysis revealed a strong association between the cuproptosis score and the infiltration level of various immune cells in the TME (Figure 9B). As described previously, we also examined immune infiltration and associated function in the high- and low-score groups. Immune cell infiltration and immune-related functional activity were significantly dysregulated between the two groups (Figures 9D,C). Moreover, we analyzed the essential markers of immunotherapy, including cytotoxic T-lymphocyte-associated antigen 4 (CTLA-4) and programmed death 1/programmed death ligand 1(PD-1/PD-L1), well-established ICP inhibitors. The expression levels of PD-1 and PD-L1 were dramatically elevated in the high cuproptosis score group, whereas the expression level of CTLA-4 did not differ significantly (Figures 9E–G).

**FIGURE 10:**
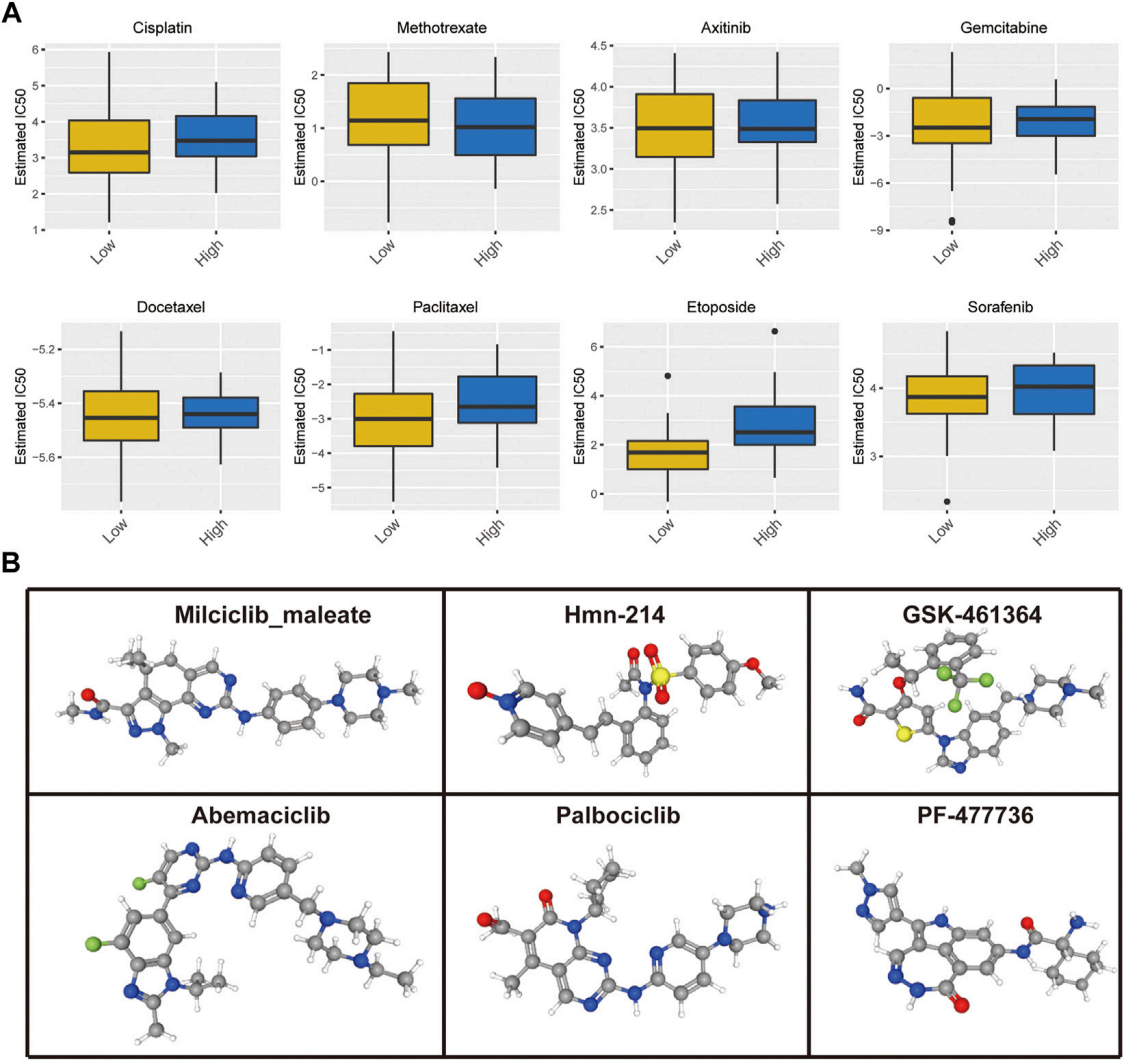
**(A)** Cuproptosis score predicts the responsiveness of OS to chemotherapy. **(B)** The 3D structure tomographs of the candidate small-molecule drugs targeting CDKN2A.

In addition, we assessed the half-maximal inhibitory concentration (IC50) values of eight distinct chemotherapeutic drugs, including cisplatin, methotrexate, axitinib, sorafenib, gemcitabine, docetaxel, paclitaxel, and etoposide, to determine the cuproptosis score and predict the likelihood of antitumor treatment response in patients with OS. The IC50 estimates of cisplatin (*p* = 0.045), paclitaxel (*p* = 0.033), and etoposide (*p* < 0.001) were significantly higher in the high cuproptosis score group than those in the low cuproptosis score group; there was no significant difference in the IC50 estimates of other drugs (Figure 10A). In the aspect of targeted therapy, the Drug Gene Interaction Database (DGIdb; https://dgidb.org/) was used to search for small molecule drugs with therapeutic effect on OS. Among them, drug experiments targeting CDKN2A have obvious advantages. 3D structural tomography of Milciclib maleate, HMN-214, GSK-461364, Abemaciclib, Palbociclib and PF-477736 were found in PubChem (https://pubchem.ncbi.nlm.nih.gov/) (Figure 10B).

### Validation of the protein levels of CDKN2A in OS and tumor-adjacent tissues

**FIGURE 11:**
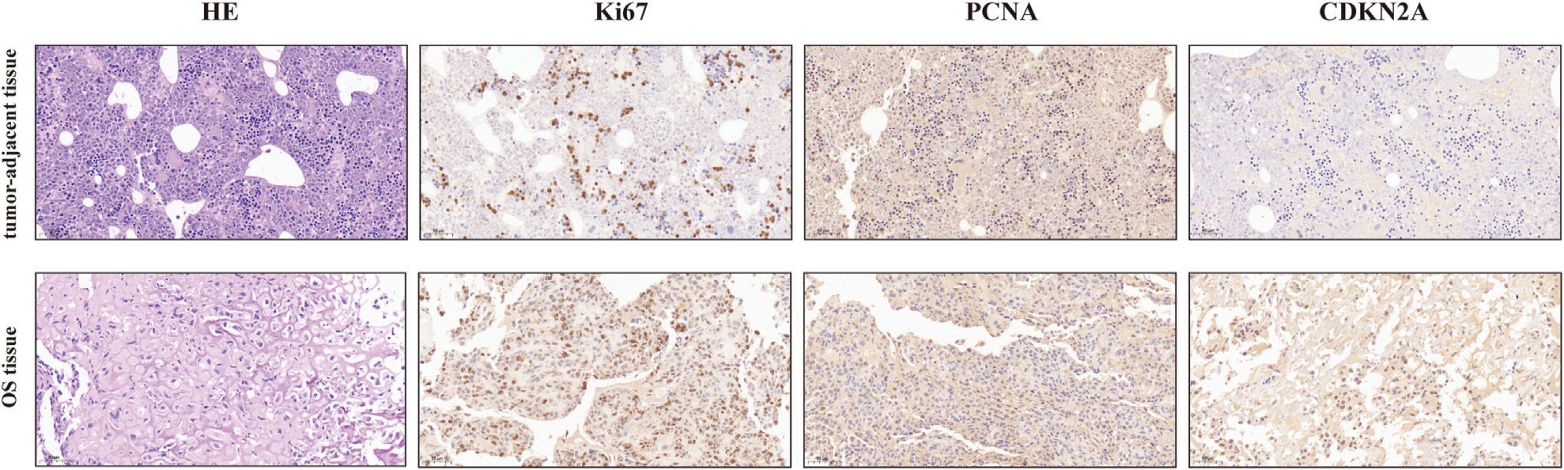
IHC analysis of ki-67, PCNA, and CDKN2A in OS and tumor-adjacent tissues (Magnification, ×200, scale bars indicated 50 μm).

The protein levels of CDKN2A in OS and tumor-adjacent tissues were detected by corresponding antibody and IgG (isotype) immunohistochemistry. The results showed that the expression of CDKN2A in osteosarcoma tissues was higher than that in tumor-adjacent tissues (Figure 11). At the same time, we also detected Ki67 and PCNA, which were related to tumor proliferation, and the results showed that the protein level of CDKN2A in OS tissues was positively correlated with ki67. This is consistent with the results of our bioinformatics analysis. These results suggest that CDKN2A may be potential targets for the diagnosis and treatment of OS patients.

## Discussion

Copper ions are indispensable enzyme-catalyzed cofactors that drive a series of important biochemical processes, including signal transduction, cell metabolism, and energy production (Ge et al., 2022). During intracellular homeostasis, intracellular free copper is typically restricted to markedly low levels, resulting in the exploration of mechanisms underlying copper transport (Shanbhag et al., 2021). The cellular copper accumulation that exceeds the threshold induces cell toxicity and eventually cell death (Cobine and Brady, 2022; Tsvetkov et al., 2022). This form of cell death is considered to result from copper-induced cytotoxicity. There is a close relationship between copper and mitochondrial respiration. More recently, this method of cell death has been termed cuproptosis (Tsvetkov et al., 2022). There is growing evidence that cuproptosis can affect tumor growth and induce tumor cell death, thus playing an indispensable role in tumor immunity and antitumor therapy (Zhang et al., 2022a; Bian et al., 2022). However, its role in the development of the TME, as well as its relationship with OS, remain unclear. Herein, we comprehensively described how cuproptosis patterns of OS are clinically significant and their relationship with the TME characteristics. In addition, a cuproptosis score system was proposed to evaluate individual cuproptosis to improve awareness of the TME and assist physicians in developing more effective immunotherapy strategies. Finally, we verified the abnormal expression of CDKN2A in OS tissues and predicted small molecule drugs targeting it.

We first explored the influence of the 10 CUGs on the survival of patients with OS. The results revealed that patients with high expression levels of CDKN2A and FDX1 had poor survival probability, whereas patients with high expression of LIAS, LIPT1, and PDHA1 had better survival probability. Using unsupervised clustering, we developed two OS molecular patterns based on the mRNA expression profiles of the CUGs. There were significant differences in clinical outcomes between the two groups. In addition to survival and clinical relevance, immune-related features are critical in cancer progression and treatment. A parallel analysis of immune cell infiltration and immune-related function was performed in the subgroups of patients with OS: CUGs subgroup, DEGs subgroup, and cuproptosis score subgroup. Notably, each subset exhibited distinct immune cell infiltration and immune function. Considering the cuproptosis scoring system, the distribution of different types of immune cells and immune functions in patients with certain scores were inconsistent. The role of cuproptosis in immune properties remains unclear, and more specific studies are needed to clarify the importance of cuproptosis in the TME. Interestingly, previous studies have extensively reported the significant effects of copper on the immune response and microenvironment of cancer. However, research on immune correlation in cuproptosis remains limited. Mitra et al. have highlighted that excessive copper exposure can lead to apoptosis and cell cycle arrest, possibly leading to immunotoxicity, thereby revealing how apoptotic pathways distinctly modulate copper-induced immunosuppression.

On the other hand, immunotherapy has great potential in the treatment of human malignant tumors (Yang, 2015; Kennedy and Salama, 2020). Immunotherapy can effectively improve the OS prognosis (Chen et al., 2021; Ogiwara et al., 2022). Studies have shown that PD-1, PD-L1, and CTLA-4 affect the progression of OS, and representative immunotherapy drugs include pembrolizumab/nivolumab (PD-1 inhibitor), ipilimumab (CTLA-4 inhibitor), and BMS-936559 (PD-L1 inhibitor) (Vilain et al., 2017; Tawbi et al., 2018). Our results revealed that expression levels of PD-1 and PD-L1 in the high-score group were significantly higher than those in the control group, while the expression levels of CTLA-4 did not differ significantly. This finding suggests that patients with high cuproptosis scores may be more sensitive to anti-PD1 and anti-PD-L1 treatment. However, copper has been previously associated with the efficacy of cancer immunotherapy. Copper was shown to upregulate PD-L1 expression and contribute to cancer immune evasion, and copper chelators could reverse this effect by promoting ubiquitin-mediated PD-L1 degradation (Voli et al., 2020). Zhou et al. have also reported that a combination of dithiolan and copper can stabilize PD-L1 expression and induce immunosuppression in hepatocellular carcinoma (Zhou et al., 2019). Furthermore, given the irreplaceable role of chemotherapy in treating OS, we assessed whether the cuproptosis score could affect chemotherapy sensitivity in patients with OS. We detected significant differences in the IC50 values of cisplatin, paclitaxel, and etoposide between the high- and low-scoring groups. In conclusion, the cuproptosis score is potentially indicative of the immunotherapy response and chemotherapy sensitivity in patients with OS. For targeted therapy, Milciclib maleate, HMN-214, GSK-461364, Abemaciclib, Palbociclib and PF-477736 have been shown to be potential targeted drugs for OS. Finally, we also found abnormal up-regulation of CDKN2A protein level in OS tissues by immunohistochemistry.

There are still some deficiencies in the study. First of all, we did not delve deeply into the mechanisms by which cuproptosis affects the clinical and molecular characteristics of OS. Furthermore, we have not been able to clarify the relationship between cuproptosis and time, and therefore we call for more in-depth studies of cuproptosis in future studies.

## Conclusion

In conclusion, we systematically analyzed the effects of cuproptosis on OS and provided a clear explanation of the broad regulatory mechanisms, clinicopathological features and prognosis of cuproptosis in TME. In addition, we demonstrated the power of CUGs as a biomarker of treatment response. Small molecule drugs that specifically target the cuproptosis pathway in OS patients were also predicted. Therefore, a comprehensive assessment of cuproptosis score in each OS patient is of great clinical importance and can be used to develop personalized treatment strategies for these patients.

## Data Availability

The datasets presented in this study can be found in online repositories. The names of the repository/repositories and accession number(s) can be found in the article/Supplementary Material.
