# Supplementary figures and images for: Comprehensive analysis of cuproptosis in immune response and prognosis of osteosarcoma
Source: Front Pharmacol. 2022 Oct 3;13:992431. doi: 10.3389/fphar.2022.992431 (PMC9573992; doi:10.3389/fphar.2022.992431)

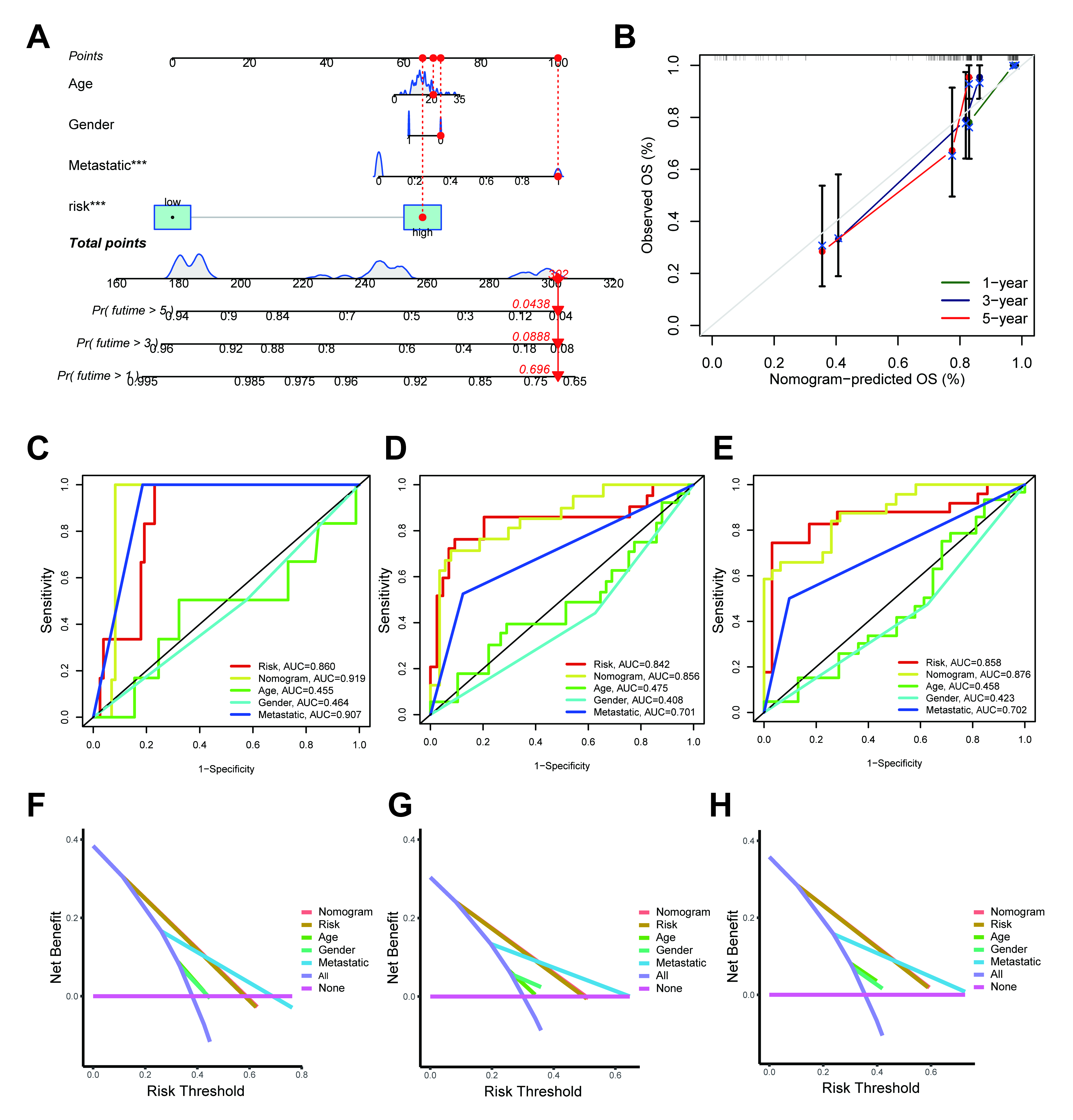

Supplement: Supplementary file 2 [file Image3.TIF]

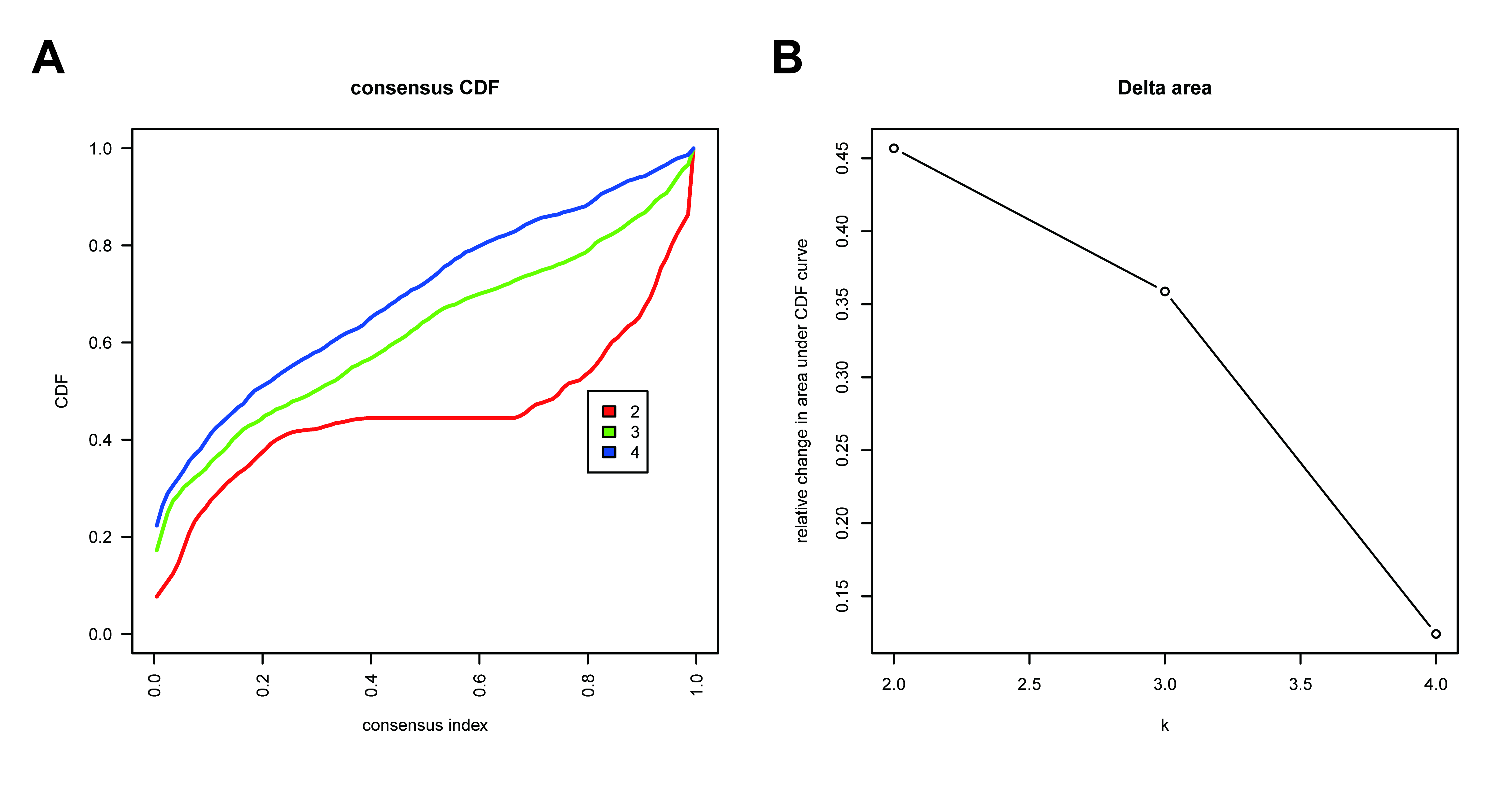

Supplement: Supplementary file 3 [file Image4.TIF]

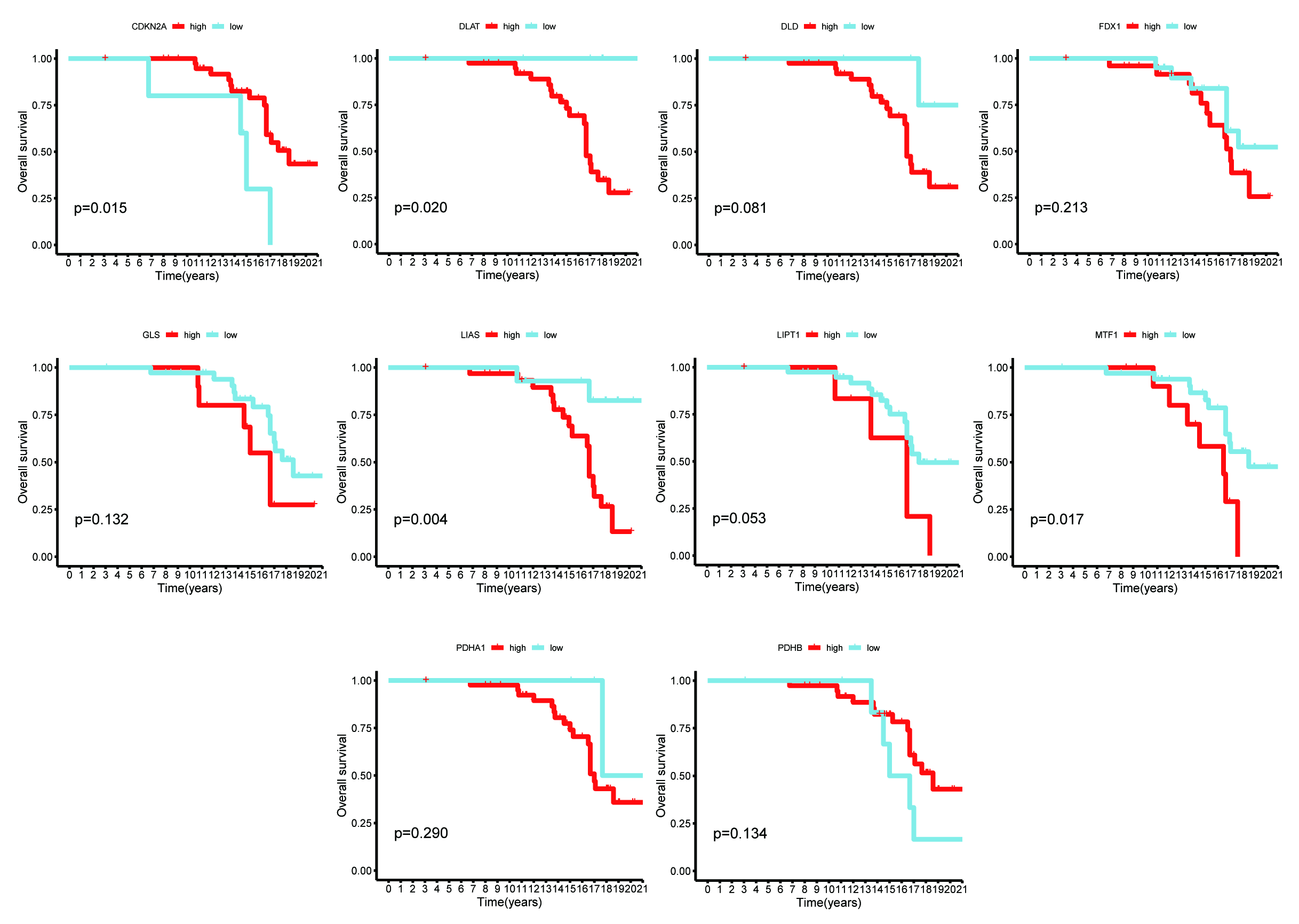

Supplement: Supplementary file 5 [file Image1.TIF]
